# Conceptualizing psychological resilience through resting-state functional MRI in a mentally healthy population: a systematic review

**DOI:** 10.3389/fnbeh.2023.1175064

**Published:** 2023-07-19

**Authors:** Alan P. L. Tai, Mei-Kei Leung, Xiujuan Geng, Way K. W. Lau

**Affiliations:** ^1^Department of Special Education and Counselling, The Education University of Hong Kong, Hong Kong, Hong Kong SAR, China; ^2^Integrated Centre for Wellbeing, The Education University of Hong Kong, Hong Kong, Hong Kong SAR, China; ^3^Bioanalytical Laboratory for Educational Sciences, The Education University of Hong Kong, Hong Kong, Hong Kong SAR, China; ^4^Department of Counselling and Psychology, Hong Kong Shue Yan University, Hong Kong, Hong Kong SAR, China; ^5^Brain and Mind Institute, The Chinese University of Hong Kong, Hong Kong, Hong Kong SAR, China; ^6^Department of Health Sciences, The Hong Kong Metropolitan University, Hong Kong, Hong Kong SAR, China

**Keywords:** psychological resilience, resting-state, functional magnetic resonance imaging (fMRI), healthy population, operational definitions of resilience, neural mechanisms

## Abstract

Conceptualizations and operational definitions of psychological resilience vary across resilience neuroimaging studies. Data on the neural features of resilience among healthy individuals has been scarce. Furthermore, findings from resting-state functional magnetic resonance imaging (fMRI) studies were inconsistent across studies. This systematic review summarized resting-state fMRI findings in different modalities from various operationally defined resilience in a mentally healthy population. The PubMed and MEDLINE databases were searched. Articles that focused on resting-state fMRI in relation to resilience, and published before 2022, were targeted. Orbitofrontal cortex, anterior cingulate cortex, insula and amygdala, were reported the most from the 19 included studies. Regions in emotional network was reported the most from the included studies. The involvement of regions like amygdala and orbitofrontal cortex indicated the relationships between emotional processing and resilience. No common brain regions or neural pathways were identified across studies. The emotional network appears to be studied the most in association with resilience. Matching fMRI modalities and operational definitions of resilience across studies are essential for meta-analysis.

## 1. Introduction

Psychological resilience (from hereafter will be referred to resilience), a critical counteracting factor of psychopathology, is defined as the ability to rebound from a difficult situation. There are multiple conceptualizations of resilience in the field of resilience research. For instance, researchers classified resilience into two separate concepts in earlier studies: state resilience and trait resilience (van der Werff et al., 2013a). State resilience was the temporary and immediate response usually measured immediately after a stress-induced task. On the other hand, trait resilience provided more substantial information, like resilience-related personality constructs, and was more stable like a personality trait van der Werff et al., 2013a). In more recent resilience research, resilience has been conceptualized as an outcome, as a positive adaptation to adversity (Peltonen et al., 2014; Kalisch et al., 2015, 2017). Put it in terms of psychopathology prevention, stress resilience is being able to recover quickly based on experience and adaptation from a stressor exposure (a trauma) (Fleshner et al., 2011). In line with this concept, resilience is further characterized as a process, a dynamic adaptation to adversity over time (Stainton et al., 2019). Change over time is the critical difference between the outcome and process models. This model implied that resilience is not a stable trait, and it can change constantly depending on the situation of the stressor. More recently, another new approach in conceptualizing resilience has been proposed by Kalisch et al. (2019), which adds different layers of protective factors to the process model. This dynamic network approach considers resilience not as an entity but as multiple resilience factors that are responsible to different aspects of lives (Kalisch et al., 2019).

A body of literature was conducted in the past decade to understand the conceptual definition of resilience and the factors that associate with it. In the early years, resilience studies focused on at-risk teens on childhood maltreatment or trauma, which were popular in child psychiatry and developmental psychology (Vernon, 2004). Experience of childhood adversity increases the risk of developing a broad range of types of psychological disorders (Green et al., 2010), while resilience plays a critical role in positively adapting under aversive environment. Later, the focus of resilience research shifted to psychopathology, such as post-traumatic stress disorder (PTSD) and other stress-induced disorders (Horn et al., 2016). The differences between patients and normal controls were the interests of resilience research during that era. Since the comparison were made between patients and normal control, disorder-specific results of resilience were typically found in this type of research. In recent years, more focuses were put on understanding the socioeconomic and neurobiology aspects of individuals, i.e., poverty and the interplay with psychological factors on resilience (Hallegatte et al., 2020; Eaton et al., 2022). The more recent resilience research has steered away from the clinical population and focused on mentally healthy population. When focusing on mentally normal people, the protective factors of resilience are the vital interest.

### 1.1. Mentally healthy participants on resilience research

Recent resilience research emphasizes the importance of focusing on investigating protective factors rather than psychopathology. In contrast, earlier literature defined resilience based on the developments of clinical cases (Vernon, 2004). Individuals exposed to a stressor (i.e., a trauma or childhood adversity) who did not develop stress-induced disorders (i.e., PTSD) were classified as a high resilience group and comparisons were made with the clinical groups. However, a group of those who did not experience any trauma and have high resilience would be missed in this line of research. Moreover, research on a particular disorder implied disorder-specific mechanisms for resilience (Kalisch et al., 2015), which cannot be generalized to the public. This effect is more evident in neurological research on resilience, as some disorders had genetically prompted neurological differences. Recent neurological and clinical research on resilience started to recruit three specific subject groups to tackle the issue of only identifying disorder-specific mechanisms (van der Werff et al., 2013b; Kennis et al., 2015; Singh et al., 2018; Whittaker et al., 2018; Jeon et al., 2020). Besides the clinical group, the other two groups were random non-clinical individuals. Those related to the clinical cases or who experienced the same trauma as the clinical group but did not develop a psychological disorder were defined as the high resilience group. In addition, a random control group was also included. When comparing the clinical group with the other two groups, the differences in the neurological results may indicate the disorder-specific mechanisms involved (van der Werff et al., 2013b; Kennis et al., 2015; Singh et al., 2018; Whittaker et al., 2018; Jeon et al., 2020). These disorder-specific mechanisms may not be related to resilience. On the other hand, resilience research can focus on the general resilience mechanisms that promote generalization to protect individuals from different stress-induced disorders by steering away from clinical patients. For example, excluding the clinical group, results only from comparing the high resilience group with the random control group would be able to provide general resilience mechanisms. In fact, a few local scholars had targeted the non-clinical population only when conducting resilience research (Kong et al., 2015; Shao et al., 2018). And there is an increasing trend to target non-clinical individuals in more recent resilience research. To access the resilience levels among non-clinical individuals, there were methodological challenges in examining resilience among non-clinical individuals.

### 1.2. Operational definitions of resilience

In resilience research, the literature adopted a number of different methodologies to operationally define resilience (van Harmelen et al., 2017; Veer et al., 2021). To access the group of non-clinical individuals, a battery of psychometric scales has been developed to assess resilience. Different self-reported questionnaires were adapted to measure the level of resilience across studies.

Windle et al. (2011) reviewed various resilience measurements. For instance, one of the most commonly used was the scale developed by Connor and Davidson: Connor Davison resilience scale (CD-RISC) (Connor and Davidson, 2003; Windle et al., 2011). The CD-RISC can assess the overall perceived resilience of oneself (Connor and Davidson, 2003). This measure scored the highest rating overall, including reliability and validity, among nineteen other resilience measures [for more details, please refer to Windle et al. (2011)]. It was well-adopted and used in the field of resilience research. One limitation of this measure is the ambiguity of the conceptual difference between CD-RISC and coping.

In addition to the CD-RISC, the State and Trait Resilience Inventory (Hiew et al., 2000), the Resilience Scale for Adults (Friborg et al., 2003), and the Brief Resilience Scale (Smith et al., 2008) are also commonly used for assessing resilience across studies. The State and Trait Resilience Inventory captured both state resilience and trait resilience separately (Hiew et al., 2000). The population targeted during the development of this measure was university students (Hiew et al., 2000). However, the conceptualization of resilience had been shifted away from classifying as state or trait binarily (Kalisch et al., 2015, 2017), which limited the generalizability of the result from this scale. The Resilience Scale for Adults and the Brief Resilience Scale were the other two out of three recommended measures after reviewing nineteen other measures on resilience besides the CD-RISC (Windle et al., 2011). Both measures scored high in reliability and validity ratings (Windle et al., 2011). The Resilience Scale for Adults assessed resilience on multiple levels (Friborg et al., 2003). This measure was ideal with the current trend of conceptualized resilience as an active dynamic adaptation to adversity (Kalisch et al., 2015, 2017). However, this measure was limited to adults only, which lacked the potential for early identification, as it cannot be generalized and measured in a young age group (Windle et al., 2011). The Brief Resilience Scale examined resilience as an outcome, meaning the ability to recover from stress (Windle et al., 2011). Most of the items from this scale focused on individual levels, while neglecting the level of family and community (Windle et al., 2011). Nevertheless, this measure was limited to access the process of achieving the outcome, including individual resources and assets.

Although these subjective measures were well-developed and well-validated, they could not avoid the subjective bias and individual differences in terms of stressors (traumas). Thus, the outcome-based measures were adopted in more recent literature. The score in this measure was calculated by the ratio of self-reported general health conditions to the perception of stress, which can minimize the individual differences in the types of stress exposures (Veer et al., 2021). With different operationalizations of resilience, each had its advantages and limitations. However, different operationally defined resilience can lead to varying interpretations of outcomes in resilience research. Given that no studies have compared these various operational definitions of resilience in the same subject, it remains unclear whether these operational definitions point to the same resilience outcome. Furthermore, subjective bias was inevitable using self-reported methods. One way to address this research question in a more objective way is to examine the common and distinct neural correlates across these various operational definitions of resilience.

### 1.3. Neural mechanism of resilience

Resilience can be viewed from a neurological perspective. Neuroimaging data can reduce subject frauds of psychological measurement and serve as compensatory tools to validate subjective measures. There were different neuroimaging tools accessible to researchers, including electroencephalography (EEG), Magnetic Resonance Imaging (MRI), functional MRI (fMRI), and functional near-infrared spectroscopy (fNIRS). EEG utilizes electrodes to measure the electrical activity of the brain (Noachtar and Rémi, 2009). Early literature found that resilient functioning was related to greater left frontal EEG activity, and greater left hemisphere EEG activity in non-maltreated children compared to maltreated children (Curtis and Cicchetti, 2007). Recent literature also revealed the relationship between negative emotions and resilience utilizing EEG data (Chen D. et al., 2018). The main drawback of EEG in neuroimaging research was the low spatial resolution, meaning the signal received from the electrodes failed to pinpoint the exact location of the activity that occurred (Noachtar and Rémi, 2009). Another one of the most used and reliable tools in this field of research is MRI, a non-radioactive and non-invasive technology that can provide clear three-dimensional anatomical images (Katti et al., 2011). MRI is mainly used for structural data, measuring the volume or size of specific brain regions (Mills et al., 2017). However, when examining the neural mechanisms of resilience, functions of different brain regions can provide more information than the structural matter. Functional connectivity and brain activation data were often the interest of resilience research by adopting the fMRI method to analyze the time series of voxel changes in the Blood-Oxygen-Level Dependent (BOLD) signals. BOLD signals allowed researchers to understand the brain functioning at a given situation or time-point when incorporated with other behavioral testing. For example, Dennison et al. (2016) found that a greater BOLD signal in the left pallidum was associated with lower depressive symptoms in maltreated youth. This study accessed behavioral data on depression symptoms and incorporated it with BOLD signals to provide said results (Dennison et al., 2016). Like fMRI, fNIRS also indirectly measures brain function by the concentration change of oxygenated and deoxygenated hemoglobin (Mehta and Parasuraman, 2013). Compared with fMRI, low spatial resolution and penetration depths are the most significant drawbacks for fNIRS, similar to EEG. Due to the purpose of this review, specific locations of functions are essential to understanding the underlying logic of resilience from a neurological perspective. Thus, this review will focus on studies of resilience using fMRI.

With the natural built-in mechanism of human beings, resilience is proposed to be quantified via neuroimaging, resulting in the development of neural features for resilience. In neuroimaging studies on resilience, different perspectives and factors were examined, like psychological and socio-environmental factors. For psychological factors of resilience, a study indicated a circuity of subgenual anterior cingulate (sgACC) to insula as a neural correlate to resilience (Shao et al., 2018). This study provided evidence that group differences were presented in the change in resting-state functional connectivity between sgACC and insula between high resilience and low resilience group (Shao et al., 2018). These regions were associated with experience and emotional regulation (van der Werff et al., 2013b), which are important resilience factors. For socio-environmental factors, recent literature has targeted social background (e.g., poverty) to be associated with resilience (Holz et al., 2020). An fMRI study found that childhood poverty was associated with less dorsolateral prefrontal cortex (dlPFC) activities during emotion regulation in adulthood (Kim et al., 2013). This area requires a prolonged maturation period and is mainly responsible for executive functioning, planning, and regulation. Also, it is one of the key areas that can predict resilience (Moreno-López et al., 2020). The studies mentioned above yielded different neurological outcomes based on the resilience factors that were being focused on and highlighted the importance of identifying the underlying neurological mechanisms for resilience. There is an existing neural model for vulnerability and resilience (Homberg and Jagiellowicz, 2022). The author of this model suggested that differential susceptibility: genes, protective factors or traits that can affect individuals to pay more attention toward positive environmental stimuli or a negative one, is linked to brain functioning; thus, become more resilient or vulnerable (Slagt et al., 2019; Homberg and Jagiellowicz, 2022). This model suggested that increased salience network (SN) activity, increased SN and default mode network (DMN) connectivity, and increased SN and central executive network (CEN) connectivity are related to attention shifting and cognitive flexibility (Homberg and Jagiellowicz, 2022). The current review aims to examine the underlying neural mechanisms of resilience in high resilient individuals. Additional information can be provided on this existing neural model of vulnerability and resilience.

### 1.4. Advantage of resting-state fMRI for resilience research

When utilizing fMRI, data can be collected through task-based or resting-state designs. In task-based designs, individuals are typically asked to complete a task, and the changes in neural activation are examined. A recent review was performed by Eaton et al. (2022) on different neuroimaging research on resilience among young people. They included eight studies that used a task-based approach (Heitzeg et al., 2008; Hanson et al., 2015; Dennison et al., 2016; Luking et al., 2018; Callaghan et al., 2019; Rodman et al., 2019; Maciejewski et al., 2020; Wymbs et al., 2020). Three of them used implicit emotion processing tasks (Heitzeg et al., 2008; Dennison et al., 2016; Wymbs et al., 2020), while others used tasks including reward processing (Hanson et al., 2015; Luking et al., 2018) and interference tasks (inhibition) (Callaghan et al., 2019; Maciejewski et al., 2020). In Eaton et al. (2022) review, they summarized the eight included task-based studies that high resilient young people are suggested to have lower amygdala responses to negative stimuli, tighter coupling of a prefrontal cortex (PFC)-amygdala circuit (Heitzeg et al., 2008; Rodman et al., 2019), and greater or normal ventral striatal activation toward positive or rewarding stimuli (Hanson et al., 2015; Luking et al., 2018). The results from amygdala and PFC-amygdala circuit were yield from studies that applied emotion tasks (Heitzeg et al., 2008; Rodman et al., 2019); whereas results from ventral striatum were from studies that applied reward processing tasks (Heitzeg et al., 2008; Rodman et al., 2019). Inconsistent tasks limited the interpretation of the neurological results relating to resilience. However, it is unclear whether these neurological results were directly related to the process of resilience or only the emotional regulation component of resilience. Moreover, there are concerns of inconsistent task difficulties among different research and variabilities in individual ability and performance (Constable, 2006). An alternative is using resting-state design that is task-free and only requires participants not to think of anything particular during the scan.

Data from resting-state fMRI is more suitable for capturing neural resilience mechanisms from the perspective of neural functions at rest, meaning trying to relax and not think of anything during the scan. Although intrinsic activity (e.g., mind wandering) is a disadvantage of utilizing data from resting-state fMRI studies (Raichle and Snyder, 2007; Finn, 2021), there is not enough existing literatures that have adopted naturistic paradigm nor a combination of both task and rest. When compared task-based fMRI to resting-state fMRI approaches, there are more concerning limitations regarding task-based fMRI approach in the field of resilience as pointed out. Past literature has shown a good utilization of resting-state fMRI in measuring brain activities and connections associated with resilience. A few studies have shown associations of resting-state connectivity of different areas and networks to resilience in PTSD patients (Rabinak et al., 2011; Yin et al., 2011). Those areas included the posterior cingulate cortex (PCC)/precuneus region and thalamus (Rabinak et al., 2011; Yin et al., 2011). Also, positive functional connections were found between the thalamus to the right medial frontal gyrus and the thalamus to the left rostral anterior cingulate cortex (ACC) (Rabinak et al., 2011; Yin et al., 2011). These areas are generally involved in emotional regulation, inhibition, and higher executive functioning. Moreover, resting-state fMRI can capture the baseline differences in neural activities and connectivity at rest without the effect of other stimuli and conditions. This is essential for identifying the potential neural markers for resilience.

It’s noteworthy to mention that there are other neurological factors that would affect resilience-dependent change in neural activities captured in resting-state fMRI, i.e., neuroendocrine and monoamines (Russo et al., 2012; Watanabe and Takeda, 2022). Activation of the hypothalamic-pituitary-adrenal (HPA) axis, one of the well-studied neuroendocrine systems, causes a widespread of hormonal and neurochemical changes, which was found to be affecting resilience (Russo et al., 2012; Lau et al., 2021). For instance, hormones like cortisol and dehydroepiandrosterone (DHEA) are released from the adrenal cortex in response to stress (Russo et al., 2012; Lau et al., 2021). The ability to restore the ratio of these two hormones back to normal after a stressful event was found to be affecting resilience (Lau et al., 2021). Moreover, a recent review had indicated that monoamines such as dopamine, serotonin, and noradrenaline were involved in resilience (Watanabe and Takeda, 2022). These monoamines were found to be affecting neural activities in brain regions that are highly associated with resilience, such as ventromedial prefrontal cortex (vmPFC), ACC, PCC, and medial prefrontal cortex (mPFC) (Kim et al., 2013; Shao et al., 2018; Eaton et al., 2022; Watanabe and Takeda, 2022). These findings provide a possible linkage in the psycho-neuro-endocrinological explanation of resilience.

In a short summary of the above neuroimaging studies, there is a research gap in exploring the underlying neural mechanisms of different operationally defined resilience. With only limited studies assessing the neural correlates of resilience among mentally healthy individuals, the protective mechanisms of resilience are still largely unknown (Waugh et al., 2008; New et al., 2009; Daniels et al., 2012; Reynaud et al., 2013; van Rooij et al., 2016; Iadipaolo et al., 2018). In addition, more objective neural markers are still needed for identifying resilience to help with prevention and intervention and developing a good model to predict individuals who may be at-risk.

### 1.5. Aims of the current study

To the best of my knowledge, there is no systematic review summarized the findings from resting-state fMRI studies from different operationally defined resilience in a mentally healthy population in the field. The most recent review by Eaton et al. (2022) examined a similar topic but only focused on youth only. Moreover, they included studies targeting protective factors and wellbeings (Eaton et al., 2022). Therefore, some of the studies they included did not have a targeting group considered as resilient. The objective of the current review is to examine the different operationally defined resilience across studies from a neurological perspective. Findings from the neural mechanisms provide insights into resilience’s underlying/core concept. The review results can contribute to the current field of studies exploring resilience by conceptualizing resilience from the neurological perspective while identifying the similarities and the differences between different operational definitions of resilience.

## 2. Methods

### 2.1. Databases and search terms

Studies were identified using the PubMed and MEDLINE databases. The following search terms were used in both databases: (Neuroimaging OR fMRI) AND (resting OR resting-state OR default mode network OR DMN OR intrinsic brain activity OR spontaneous brain activity) AND resilience. Only human research was included in this review. The search was completed on 31st December 2021. A total of 19 articles were included in this review based on a list of inclusion and exclusion criteria.

### 2.2. Study selection

**FIGURE 1:**
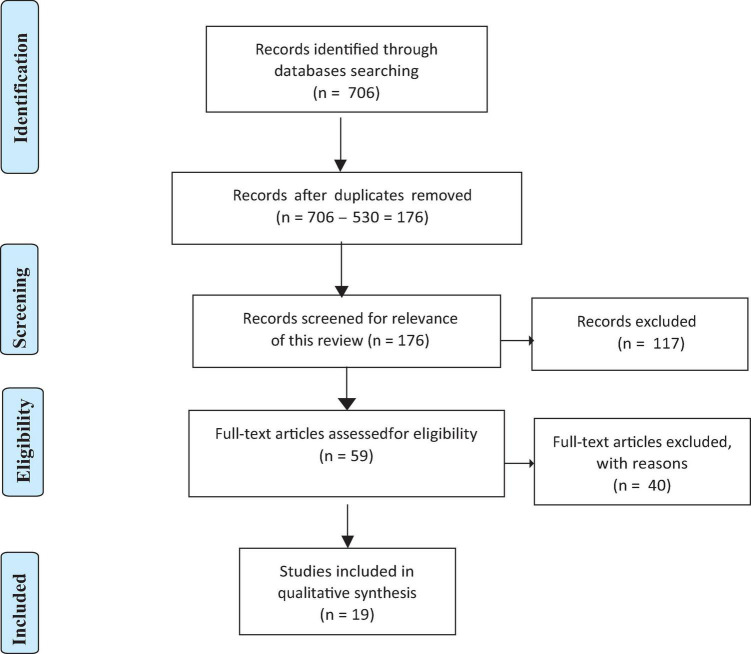
PRISMA flowchart of the study selection process.

A list of inclusion and exclusion criteria was predetermined prior to searching for eligible articles. The inclusion criteria included: (1) original peer-reviewed research; (2) having a group of participants was considered high resilience (not the healthy control group) or operationally defined resilience, i.e., quantified resilience by a resilience questionnaire score; and (3) using resting-state fMRI. In addition, any ineligible article type (i.e., review articles, conference proceedings, editorial, commentary, perspective, book chapter, book review, and dissertation), and any non-English articles were excluded. Moreover, articles that only targeted clinical participants were also excluded. See Figure 1 for the Preferred Reporting Items for Systematic Reviews and Meta-Analyses (PRISMA) flow of study selection (Page et al., 2021).

### 2.3. Search strategy

The initial search hits were recorded (*N* = 706) and sent to the reference manager (EndNote), where 530 duplicates were removed (*N* = 706–530 = 176). Then, two authors completed the abstract and full-text screening individually based on the predetermined inclusion and exclusion criteria. They cross-checked the results after both had identified the final numbers of included articles, resulting in a total of 19 articles included for this review.

### 2.4. Data extraction and replicability assessments

The 19 papers included in this review were organized and scrutinized, with a replicability assessment performed on the quality of the imaging data. Extracted data included (1) basic demographic of the participants (i.e., age and gender, etc.); (2) operational definitions of resilience (how did the article measure resilience); (3) fMRI scanning details (i.e., scan duration, eye-open/eye-close condition, etc.); (4) pre-processing steps of imaging data (i.e., motion correction and control, etc.); (5) modality used for imaging analyses (local activation or long-range functional connectivity); (6) regions-of-interests (ROIs; if any); (7) main results and resultant brain regions; and (8) control variables. Table 1 lists some of the extracted data for each of the articles. Replicability assessment was conducted based on the pre-processing steps taken for the imaging data and the scanning conditions. It is crucial to consider these aspects in neuroimaging analyses as different processing steps and different scanning conditions may yield different results. Each processing step and better scanning conditions will grant one point to the study, and the sum of points is the research’s replicability. Table 2 lists all the criteria included in the replicability assessment. Higher scores from this assessment suggested higher replicability of the research.

**TABLE 1:** Demographic of studies.

References	Sample size (N, M:F)		Age (Mean ± SD, Range)		Design	Operational definition of resilience	Whole brain/ROI analyses	Region-of-interest (ROI)
Shi et al., 2021	68 (47:21)*		21.54 ± 1.16 (19–28)		Correlation study	25-items Resilience Questionnaire	Whole brain	NA
Wang et al., 2020	231 (110:121)*		18.48 ± 0.54 (16–20)		Correlation study	Connor-Davidson Resilience Scale (CD-RISC)	Whole brain	NA
Kong et al., 2018	100 (42:58)*		20.86 ± 2.01 (18–26)		Correlation study	CD-RISC	Whole brain	NA
Fujisawa et al., 2015	30 (10:20)*		21.9 ± 3.4 (18–48)		Correlation study	Posttraumatic Growth Inventory (PTGI) score	Whole brain	NA
Kong et al., 2015	276 (127:149)*		21.57 ± 1.01 (18–25)		Correlation study	CD-RISC	Whole brain	NA
Miyagi et al., 2020	89 (59:30)*		32.1 ± 13.6 (18–68)		Correlation study	CD-RISC	Whole brain	NA
Shi et al., 2019	212 (97:115)*		22.3 ± 1.49 (19–27)		Correlation study	25-items Resilience Questionnaire	ROI	Bilateral insula, rostral anterior cingulate cortex (rACC), dorsal anterior cingulate cortex (dACC), left orbitofrontal gyrus (OFC) and bilateral dorsolateral prefrontal cortex (dlPFC)
Santarnecchi et al., 2018	102 (35:67)*		27 ± 9 (NS)		Correlation study	COPE–Nuova Versione Italiana (COPE-NVI), an Italian version of the “Coping Orientation to the Problems Experienced”	ROI	Anterior cingulate cortex (ACC), left frontopolar cortex, and left angular gyrus
Kilpatrick et al., 2015	82 (36:46)*		31.3 ± 1.5 (20–52)		Correlation study	Resilient personality scores	ROI	Seed at 20 regions in salience network (SN) and default mode network (DMN)
Uchida et al., 2015	62 (30:32)*		22.3 ± 1.6 (NS)		Correlation study	Score from Reappraisal task	ROI	Left and right anatomical amygdalae, left and right dlPFC, DMN seeds as medial prefrontal cortex (MPFC), posterior cingulate cortex (PCC), and right/left parietal (RLP/LLP)
	**High resilience group**	**Control group**	**High resilience group**	**Control group**		**Operational definition of high resilient group**		
Jeon et al., 2020	22 (7:15)	40 (10:30)	31.55 ± 8.22 (NS)	34.8 ± 11.65 (NS)	Group-comparison	Trauma-exposed healthy participants	ROI	Bilateral thalamus
Jeong et al., 2019	98 (90:8)	98 (91:7)	40.9 ± 7.8 (NS)	41.3 ± 10.4 (NS)			ROI	dACC, bilateral anterior insula, vmPFC, bilateral amygdala and hippocampus
Hirshfeld-Becker et al., 2019	15 (11:4)	8 (NS*)	10.9 ± 1.51 (12–18)	NS** (12–18)	Group-comparison	At-risk (offspring of parents with a lifetime history of MDD) participants	ROI	Separated by occupation (firefighter)
Shao et al., 2018	10 (8:2)	10 (8:2)	22.4 ± 1.14 (18–30)	21.2 ± 0.98 (18–30)	Group-comparison	CD-RISC	ROI	Bilateral sgACC
Singh et al., 2018	39 (18:21)	39 (15:24)	13.93 ± 2.38 (8–17)	13.85 ± 2.45 (8–17)	Group-comparison	At-risk (offspring of parents with depression) participants	ROI	Bilateral amygdala and bilateral nucleus accumbens (Nacc)
Whittaker et al., 2018	30 (12:18)	23 (9:14)	46.03 ± 6.94 (NS)	44 ± 4.48 (NS)	Group-comparison	At-risk (non-affected siblings of patients with BD) participants	ROI	Bilateral Nacc
Kennis et al., 2015	25 (25:0)	25 (25:0)	36.04 ± 10.15 (21–57)	34.16 ± 9.32 (21–57)	Group-comparison	Veterans without PTSD	ROI	Five bilateral seed points in the ACC were selected: Caudal, Dorsal, Rostral, Perigenual and Subgenual
Singh et al., 2014	24 (8:16)	25 (10:15)	12.25 ± 3.03 (8–17)	11.56 ± 2.29 (8–17)	Group-comparison	Healthy offspring of a parent with BD	ROI	The dorsal and ventral DMN, bilateral executive control (ECN) networks, left and right amygdala, left and right Ventrolateral prefrontal cortex (VLPFC), and the subgenual ACC
van der Werff et al., 2013b	11 (8:3)	11 (8:3)	40.36 ± 10.94 (NS)	40.45 ± 9.47 (NS)	Group-comparison	Experienced childhood maltreatment but scored negative on any DSM-IV axis-1 disorder	ROI	Left and right amygdala for limbic network, left and right dACC for salience network, PCC for the DMN and left mPFC

NA, not applicable; N, Sample size; F, female; M, male; SD, standard deviation; NS, not stated.

*Targeted participants are healthy individuals.

**Paper did not state the data for follow-up control sub-group.

**TABLE 2:** Replicability assessment on studies.

References	Eye open/eye close	Physiological Regressors		Motion correction and control				Image distortion correction using field map	Normali-zation	Using non-linear registration to EPI	Scores*
		**WM**	**CSF**	**Despik**	**Head motion regression**	**Scrub**	**Head motional control in scanner**		**With T1 image**		
Wang et al., 2020	Eye close				X						2
Kong et al., 2018	Eye close				X				X	X	4
Fujisawa et al., 2015	Eye close	X	X		X				X	X	6
Kong et al., 2015	Eye close				X		X		X	X	5
Shi et al., 2021	NS	X	X		X						3
Miyagi et al., 2020	Eye open with fixation	X	X		X	X	X	X	X	X	9
Shi et al., 2019	Eye close	X	X		X						4
Santarnecchi et al., 2018	Eye open with fixation				X		X		X	X	5
Kilpatrick et al., 2015	Eye close								X		2
Uchida et al., 2015	Eye open without fixation	X	X		X						3
Jeon et al., 2020	NS				X	X	X		X		4
Jeong et al., 2019	Eye close	X	X		X	X			X		6
Hirshfeld-Becker et al., 2019	Eye open without fixation				X						1
Shao et al., 2018	Eye open	X	X		X	X			X		6
Singh et al., 2018	NS	X	X			X			X		4
Whittaker et al., 2018	Eye close	X		X	X	X				X	6
Kennis et al., 2015	Eye open with fixation	X	X						X		4
Singh et al., 2014	Eye close	X	X		X				X		5
van der Werff et al., 2013b	Eye close	X	X		X				X		5

GM, gray matter; WM, white matter; CSF, cerebrospinal fluid.

*Calculated by adding 1 point if the study conducted and reported the preprocessing steps, and eye close or eye open with fixation during scans.

Included studies were divided into two groups based on the study designs: correlational studies and comparison studies. In the field of neuroimaging research on resilience, there are various types of operational definitions of resilience used based on the purposes of the study. A universally accepted operational definition of resilience is still lacking; therefore, a grouping strategy based on the operational definition of resilience for this review is not viable. To better organize and summarize all the findings from the included studies, grouping based on study design was adopted for this review. Only studies with correlational design (correlate resilience with neural mechanisms) would be included in the group of correlational studies. On the other hand, studies in comparison groups would compare results between an operationally defined high resilience group and random controls. Due to the various types of operational definitions of resilience, neural results from the included studies were scattered. A grouping based on the network systems of the reported neural regions was adopted in this review. The primary focus of the network systems would be on cognition and emotion, as these two domains were proven to be significantly related to resilience (Kalisch et al., 2015, 2019). After data extraction and replicability assessments, results from each included study were further summarized into different networks, including cognitive and emotional networks (Catalino et al., 2020).

### 2.5. Preliminary meta-analysis

For the included studies, activation likelihood estimation (ALE) meta-analysis was performed to examine the common neural features of resilience among healthy individuals. Limited number of studies were included, and the fMRI modalities in these included studies were diverse. Studies of each group would be further considered to the following inclusion criterion to be included for the preliminary meta-analysis. Studies were included only if four or more studies were adopting the same fMRI modalities: local or ROIs to ROIs, whole-brain or same *a priori* ROIs. Based on these criteria, only four studies (Fujisawa et al., 2015; Kong et al., 2015, 2018; Wang et al., 2020) that examined the local activation with a whole-brain approach were included. The coordinate-based ALE meta-analysis was conducted by GingerALE version 3.0.2 (The BrainMap Database^1^; San Antonio, TX, USA). The reported coordinates in MNI space were imported into the software. The ALE image was thresholded using uncorrected *p* < 0.001 and a cluster-level inference threshold of *p* < 0.05 with 5,000 permutations to better control for the false-positive rates.

## 3. Results

### 3.1. Overview

In this review, a total of 19 studies were included based on the inclusion and exclusion criteria. The included studies were published between 2013 and 2021. The age of the subjects included in the 19 studies ranged from 10.9 to 44 years. Two studies only recruited male participants (Kennis et al., 2015; Hemington et al., 2018). Two studies had exact gender matched across groups (van der Werff et al., 2013b; Shao et al., 2018). All studies with pre-selected region-of-interest (ROI) are summarized in Table 3. The average score of the replicability assessment is 4.42 (SD = 1.84, total score = 10). Only one paper scored higher than 6 (score = 9) (Miyagi et al., 2020). Other papers scored lower than 6. Due to the lack of a universally accepted operational definition of resilience, included studies were divided into two groups based on the study designs: correlational studies and comparison studies. Studies with the same study design are reported together.

**TABLE 3:** Region-of-interest (ROI) for all included ROI-based studies.

Corresponding network	Region-of-interest	Counts
Emotional network	Amygdala	6 (van der Werff et al., 2013b; Singh et al., 2014, 2018; Uchida et al., 2015; Hirshfeld-Becker et al., 2019; Jeong et al., 2019)
Emotional network	Subgenual anterior cingulate cortex	4 (Singh et al., 2014; Kennis et al., 2015; Shao et al., 2018; Hirshfeld-Becker et al., 2019)
Salience Network	Dorsal anterior cingulate cortex	4 (van der Werff et al., 2013b; Kennis et al., 2015; Jeong et al., 2019; Shi et al., 2019)
Default Mode Network	Medial prefrontal cortex	3 [van der Werff et al., 2013b (left); Uchida et al., 2015; Hirshfeld-Becker et al., 2019]
Default Mode Network	Posterior cingulate cortex	3 (van der Werff et al., 2013b; Uchida et al., 2015; Hirshfeld-Becker et al., 2019)
Central Executive Network	Dorsolateral prefrontal cortex	3 (Uchida et al., 2015; Hirshfeld-Becker et al., 2019; Shi et al., 2019)
Emotional Network	Nucleus accumbens	2 (Singh et al., 2018; Whittaker et al., 2018)
Salience Network	Rostral anterior cingulate cortex	2 (Kennis et al., 2015; Shi et al., 2019)
Default Mode Network	Ventromedial prefrontal cortex	2 (Singh et al., 2014; Jeong et al., 2019)
Salience Network	Anterior insula	1 (Jeong et al., 2019)
Emotional Network	Thalamus	1 (Jeon et al., 2020)
Salience Network	Insula	1 (Shi et al., 2019)
Memory Network	Hippocampus	1 (Jeong et al., 2019)
Salience Network	Caudal anterior cingulate cortex	1 (Kennis et al., 2015)
Salience Network	Perigenual anterior cingulate cortex	1 (Kennis et al., 2015)
Emotional Network	Orbitofrontal cortex	1 [ Shi et al., 2019 (left)]
Salience Network	Anterior cingulate cortex	1 (Santarnecchi et al., 2018)
Central Executive Network	Frontopolar cortex	1 [ Santarnecchi et al., 2018 (left)]
Memory Network	Angular gyrus	1 [ Santarnecchi et al., 2018 (left)]
Central Executive Network	Parietal cortex	1 (Uchida et al., 2015)

### 3.2. Correlational studies

Ten studies adopted a single group correlational design, in which scores of resilience measured by different scales were correlated with resting-state fMRI data. Within these ten studies, three studies explored the correlation between resilience and local activation of the brain (Kong et al., 2015; Wang et al., 2020); while five studies looked at the correlation between resilience and long-range functional connectivity (Kilpatrick et al., 2015; Uchida et al., 2015; Santarnecchi et al., 2018; Shi et al., 2019, 2021; Miyagi et al., 2020). One study explored both local activation and long-range functional connectivity (Fujisawa et al., 2015). The average score of the replicability assessment among the studies in the group was similar to the overall average (*M* = 4.3. SD = 2.11). Miyagi et al. (2020) conducted the highest scored study, which focused on the correlation between long-range functional connectivity and resilience. The associations of resilience with local brain activation and long-range functional connectivity among the ten included studies in this group were summarized below.

#### 3.2.1. Local activation

**FIGURE 2:**
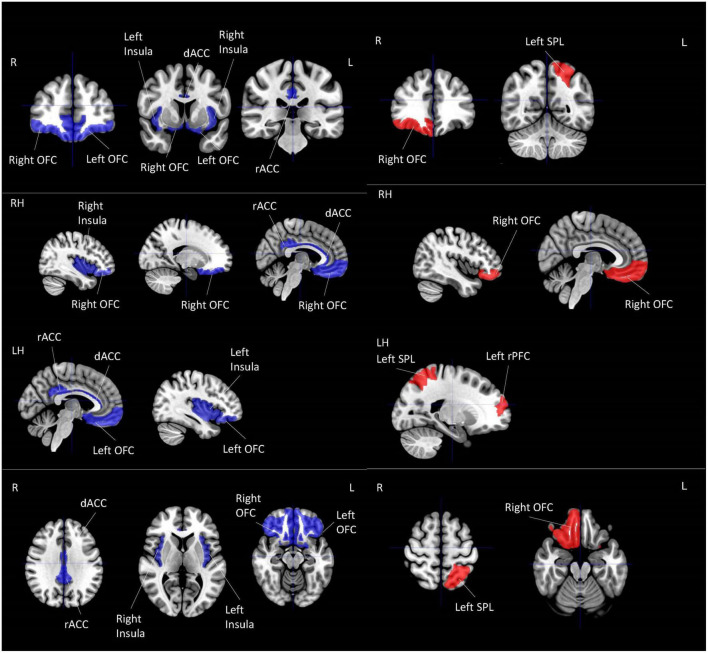
Local activation for correlational studies. Red represents positive correlation with resilience. Blue represents negative correlation with resilience. All highlighted regions showed in the figure are only used for approximate representation of the reported regions. It does not represent exact coordinates nor clusters of the reported results. R, Right; L, Left; RH, right hemisphere; LH, left hemisphere; OFC, orbitofrontal cortex; SPL, superior parietal lobule; PFC, prefrontal cortex; dACC, dorsal anterior cingulate cortex; rACC, rostral anterior cingulate cortex.

All of the four studies which explored the correlation between resilience and local activation of the brain adopted a whole-brain approach (Table 4; Figure 2). Each of these four studies utilized a different MRI modality, including amplitude of low-frequency fluctuation (ALFF) (Wang et al., 2020), fractional ALFF (fALFF) (Kong et al., 2018), group spatial independent component analysis (gICA) (Fujisawa et al., 2015), and regional homogeneity (Reho) (Kong et al., 2015). Three out of four studies used the CD-RISC in terms of the operational definition of resilience (Kong et al., 2015, 2018; Wang et al., 2020). The remaining study adopted the Posttraumatic Growth Inventory (PTGI) score for measuring resilience (Fujisawa et al., 2015). Two studies found the activation of the orbitofrontal cortex (OFC) was correlated with resilience measured by the CD-RISC (Kong et al., 2018; Wang et al., 2020). This cortical area is included in the emotional network (EN) (Catalino et al., 2020) that is responsible for emotional processing. Fujisawa et al. (2015) found that resilience was positively correlated with local activation in the left rostral prefrontal cortex and left superior parietal lobule (SPL). These two areas were included in the DMN and CEN, respectively (Catalino et al., 2020). Last but not least, the brain areas found in Kong et al. (2015) were all included in SN but in an opposite direction of the correlation when compared with the findings from Fujisawa et al. (2015). Kong et al. (2015) found that the local activation of right dorsal ACC (dACC), right rostral ACC (rACC), and bilateral insula were negatively correlated with resilience. The preliminary coordinate-based ALE meta-analysis was conducted with these four studies. No common clusters were found.

**TABLE 4:** Local activation for correlational studies.

References	Operational definition of resilience	MRI modality	Resultant brain area/regions			Replicability score***
			**Positively correlate with resilience ⬈**	**Negatively correlate with resilience ⬊**	**Network related**	
Wang et al., 2020	Connor-Davidson Resilience Scale (CD-RISC)	Amplitude of low-frequency fluctuation (ALFF)	Right Orbitofrontal Cortex for males	Right Orbitofrontal Cortex for females	Emotional network	2
Kong et al., 2018	CD-RISC	Fractional ALFF (fALFF)		Left Orbitofrontal Cortex	Emotional network	4
Fujisawa et al., 2015 **	Posttraumatic Growth Inventory (PTGI) score	Group spatial independent component analysis (ICA)	Left Rostral Prefrontal Cortex		Default mode network	6
			Left Superior Parietal Lobule (SPL)		Central executive network	
Kong et al., 2015	CD-RISC	Regional homogeneity (Reho)		Right Dorsal ACC (dACC)	Salience network	5
				Right Rostral ACC (rACC)	Salience network	
				Left and Right Insula	Salience network	
						Maximum score = 10

*All studies in this table adopted a whole-brain analysis approach.

**This paper performed analyses on both local activation and long-range connectivity. For the result of long-range connectivity, please refer to Table 5.

***A higher score indicates more information was provided in controlling imaging quality. See Table 2 for calculation components and process. All papers stated in this table used a whole-brain analysis approach.

#### 3.2.2. Long-range functional connectivity

**FIGURE 3:**
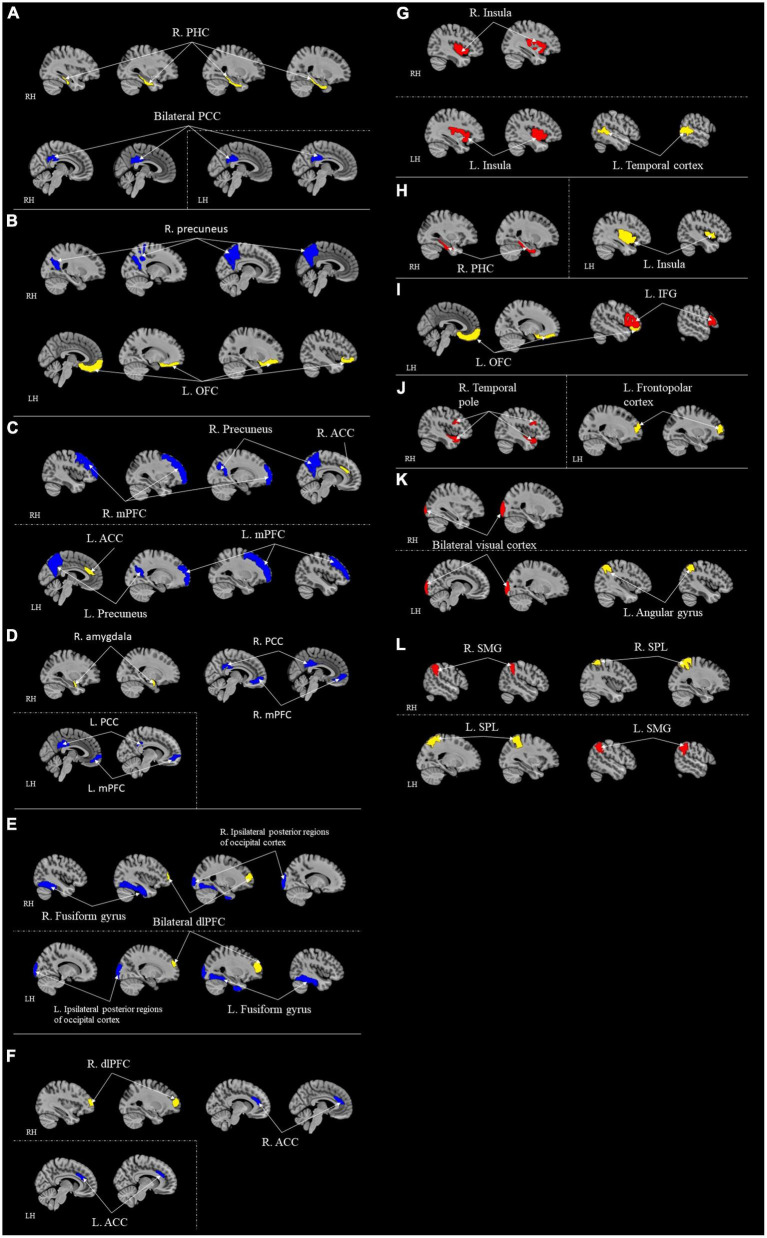
ROI-to-ROI functional connectivity for correlational studies. Red represents positive correlation with resilience. Blue represents negative correlation with resilience. Yellow represents the seed regions. Each sub-section represents connectivity from a same seed: **(A)** R. PHC (Miyagi et al., 2020); **(B)** L. OFC (Shi et al., 2019); **(C)** Bilateral ACC (Santarnecchi et al., 2018); **(D)** R. Amygdala (Uchida et al., 2015); **(E)** Bilateral dlPFC (Uchida et al., 2015); **(F)** R. dlPFC (Uchida et al., 2015); **(G)** Temporal cortex (Shi et al., 2021); **(H)** L. Insula (Shi et al., 2019); **(I)** L. OFC (Shi et al., 2019); **(J)** L. Frontopolar cortex (Santarnecchi et al., 2018); **(K)** Angular gyrus (Santarnecchi et al., 2018); **(L)** Bilateral SPL (Santarnecchi et al., 2018). Kilpatrick et al. (2015) contained too many regions that it is difficult and complicated to represent in figures; thus, it is excluded from this figure. All highlighted regions showed in the figure are only used for approximate representation of the reported regions. It does not represent exact coordinates nor clusters of the reported results. R, Right; L, Left; RH, right hemisphere; LH, left hemisphere; PHC, parahippocampal cortex; RSC, retrosplenial cortex; PCC, posterior cingulate cortex; IFG, inferior frontal gyrus; ACC, anterior cingulate cortex; OFC, orbitofrontal cortex; mPFC, medial prefrontal cortex; dlPFC, dorsolateral prefrontal cortex; PHG, parahippocampus gyrus; SMG, supramarginal gyrus; SPL, superior parietal lobule.

Including Fujisawa et al. (2015) study, seven studies examined the long-range functional connectivity and its correlation with resilience (Table 5; Figure 3). A study examined functional connectivity while weights in independent component analysis (ICA) value and conducted a partial least squares (PLS) multivariate analysis (Kilpatrick et al., 2015). Other studies adopted different modalities, including ROI-to-ROI functional connectivity (Miyagi et al., 2020; Shi et al., 2021), ROIs-based functional connectivity (Shi et al., 2019), voxel-wise connectivity analysis (Fujisawa et al., 2015; Santarnecchi et al., 2018), and seed-to-voxel correlations (Uchida et al., 2015). All seven studies adopted different measures of operationally defined resilience, including PTGI scores (Fujisawa et al., 2015), CD-RISC (Miyagi et al., 2020), 25-items resilience questionnaire (Shi et al., 2019, 2021), coping orientation to the problems experienced (Santarnecchi et al., 2018), resilience personality scores (Kilpatrick et al., 2015), and scores from the reappraisal task (Uchida et al., 2015).

**TABLE 5:** ROI-to-ROI functional connectivity for correlational studies.

References	Operational definition of resilience	Seed (ROIs/whole brain)	Target regions (ROIs/whole brain)	Resultant brain area/regions (seed to sink)		Networks related						Replicability score*
				**Positively correlate with resilience ⬈**	**Negatively correlate with resilience ⬊**	**DMN**	**CEN**	**SN**	**AN**	**MN**	**EN**	
Shi et al., 2021	25-items Resilience Questionnaire	Whole brain [Dosenbach_160, 160 ROI (Dosenbach et al., 2010)]	Whole brain [Dosenbach_160, 160 ROI (Dosenbach et al., 2010)]	Temporal cortex and insula		✓		✓				3
Miyagi et al., 2020	Connor-Davidson Resilience Scale (CD-RISC)	10 ROIs in the dorsal DMN: the medial prefrontal cortex/anterior cingulate cortex/orbitofrontal cortex (MPFC/ACC/OFC), left angular gyrus (AG l), right superior frontal gyrus (SFG r), posterior cingulate cortex/precuneus (PCC/Prec), midcingulate cortex (MCC), right angular gyrus (AGr), left and right thalamus (Thallr), left hippocampus (Hipp l), and right hippocampus (Hipp r)., and nine ROIs in the ventral DMN: the left retrosplenial cortex/posterior cingulate cortex (RSC/PCC l), left middle frontal gyrus (MFG l), left parahippocampal cortex (PaHC l), left middle occipital gyrus (MOG l), right retrosplenial cortex/posterior cingulate cortex (RSC/PCC r), precuneus (Prec), right superior frontal gyrus/middle frontal gyrus (SFG/MFG r), right parahippocampal gyrus (PaHC r), right angular gyrus/middle occipital gyrus (AG/MOG r), and the right cerebellar lobule IX (Lobule9 r). Total of 19 ROIs	Same 10 ROIs in the dorsal DMN, and same nine ROIs in the ventral DMN. Total of 19 ROIs		Right parahippocampal cortex (PHC) and left retrosplenial cortex/posterior cingulate cortex (RSC/PCC)	✓				✓		9
Shi et al., 2019	25-items Resilience Questionnaire	Predefined ROIs: Bilateral insula, rostral anterior cingulate cortex (rACC), dorsal anterior cingulate cortex (dACC), left orbitofrontal gyrus (OFC) and bilateral dorsolateral prefrontal cortex (dlPFC)	Whole brain (seed-to-voxel)	Left insula and the right parahippocampus gyrus (PHG)	Left orbitofrontal gyrus (OFC) and the right precuneus		✓	✓		✓	✓	4
				Left OFC and the left inferior frontal gyrus (IFG)							✓	
Santarnecchi et al., 2018	COPE–Nuova Versione Italiana (COPE-NVI), an Italian version of the “Coping Orientation to the Problems Experienced”	Whole-brain analysis that used a functionally defined atlas by Shen et al. (2013) with 184 ROIs to find the following seeds: Anterior cingulate cortex (ACC), left frontopolar cortex, and left angular gyrus	Whole brain (seed-to-voxel)	Left frontopolar cortex and right temporal pole	Anterior cingulate cortex (ACC) and medial prefrontal and precuneus cortices bilaterally	✓	✓	✓				5
				Left angular gyrus and visual cortex bilaterally (occipital pole)					✓	✓		
Fujisawa et al., 2015	Posttraumatic Growth Inventory (PTGI) score	Medial prefrontal cortex (mPFC), posterior cingulate cortex (PCC), dorsolateral PFC, dorsolateral PCC, dorsal anterior cingulate cortex (ACC), insular cortex, superior temporal gyrus, inferior frontal gyrus, precentral gurus, supplementary motor area, and occipital lobe	Medial prefrontal cortex (mPFC), posterior cingulate cortex (PCC), dorsolateral PFC, dorsolateral PCC, dorsal anterior cingulate cortex (ACC), insular cortex, superior temporal gyrus, inferior frontal gyrus, precentral gurus, supplementary motor area, and occipital lobe	Superior parietal lobule (SPL) and Supramarginal gyrus (SMG)			✓					6
Uchida et al., 2015	Score from Reappraisal task	Left and right anatomical amygdalae, left and right dlPFC, DMN seeds as medial prefrontal cortex (MPFC), posterior cingulate cortex (PCC), and right/left parietal (RLP/LLP)	Whole brain (seed-to-voxel)		Right amygdala to medial prefrontal cortex (MPFC) and the PCC	✓	✓		✓		✓	3
					Bilateral dorsolateral prefrontal cortex (dlPFC) to ipsilateral posterior regions of occipital cortex and fusiform gyrus		✓		✓			
					Right dlPFC and ACC		✓		✓			
Kilpatrick et al., 2015	Resilient personality scores	20 regions in salience network (SN): The identified component included anterior and posterior insula, rolandic operculum, ACC, midcingulate cortex as well as some portions of the dorsolateral prefrontal cortex, and default mode network (DMN): the medial prefrontal cortex (middle and superior frontal gyri), ACC, the posterior cingulate cortex, retrosplenial cortex, precuneus, the superior temporal sulcus (STS; middle and superior temporal gyri) and tempoparietal junction (TPJ; angular gyrus and supramarginal gyri)	20 regions in SN and DMN, same as seed	Regions in DMN and regions in SN: pregenual anterior cingulate cortex/anterior midcingulate Cortex and the STS: middle and superior temporal gyri and TPJ: angular gyrus and supramarginal gyri; retrosplenial cortex and SN		✓		✓				2
									Maximum score = 10			

DMN, Default Mode Network; FN, Frontoparietal Network; SN, Salience Network; AN, Attention Network; MN, Memory Network; EN, Emotional Network.

*A higher score indicates more information was provided in controlling imaging quality. See Table 2 for calculation components and process.

Among these seven studies, only three adopted a whole-brain approach (Fujisawa et al., 2015; Miyagi et al., 2020; Shi et al., 2021). Other four studies chose different *a priori* ROIs for their analyses (Kilpatrick et al., 2015; Uchida et al., 2015; Santarnecchi et al., 2018; Miyagi et al., 2020) (refer to Table 5 for ROIs details). Miyagi et al. (2020) took seven different *a priori* ROIs. Santarnecchi et al. (2018) chose three regions for *a priori* ROIs. Eight *a priori* ROIs were adopted in Uchida et al. (2015) study. Kilpatrick et al. (2015) predetermined 20 different regions as their *a priori* ROIs. The ACC and mPFC are the two of the most common regions that more than one study chose to be *a priori* ROIs.

Following were the specific seed to target functional connectivity findings from each included study in this group. Shi et al. (2021) found that the functional connectivity between temporal cortex and insula was positively correlated to resilience. Fujisawa et al. (2015) found that functional connectivity from superior parietal lobule to supramarginal gyrus was positively correlated with resilience. Uchida et al. (2015) found the functional connectivity from the right amygdala seed to the mPFC and the PCC, from bilateral dlPFC to ipsilateral posterior regions of occipital cortex and fusiform gyrus, and from right dlPFC to ACC were negatively correlated with resilience. Shi et al. (2019) found the function connectivity from the left insula to the right parahippocampus gyrus (PHG), and from the left OFC and the left inferior frontal gyrus (IFG) were positively correlated with resilience. This study also found that functional connectivity from the left OFC to the right precuneus was negatively correlated with resilience (Shi et al., 2019). Santarnecchi et al. (2018) found the connectivity between the left frontopolar cortex and right temporal pole and left angular gyrus and visual cortex bilaterally (occipital pole) were positively correlated with resilience. A negative correlation was also found in this study between resilience and the connectivity between ACC and medial prefrontal and precuneus cortices bilaterally (Santarnecchi et al., 2018). Kilpatrick et al. (2015) found that the functional connectivity between regions in the DMN and the SN were positively correlated with resilience.

After summarizing the results into network levels, two studies found resultant areas included in the CEN (Fujisawa et al., 2015; Uchida et al., 2015). Five studies found resulting regions included in DMN (Kilpatrick et al., 2015; Uchida et al., 2015; Santarnecchi et al., 2018; Miyagi et al., 2020; Shi et al., 2021). Four other studies found resulting regions included in SN (Uchida et al., 2015; Santarnecchi et al., 2018; Shi et al., 2019, 2021). Attention network (AN) was also involved in two studies (Uchida et al., 2015; Santarnecchi et al., 2018). Three studies found resultant regions included in the memory network (MN) (Santarnecchi et al., 2018; Shi et al., 2019; Miyagi et al., 2020). Two studies found resultant regions included in the EN (Uchida et al., 2015; Shi et al., 2019). MN included regions like hippocampal formations, the cingulate cortex, and the angular gyrus responsible for recognition memory functions, encoding, and vision-related memory processing (Catalino et al., 2020). Notably, CEN is involved in the most connectivity compared to other networks (five pathways).

### 3.3. Comparison studies

**FIGURE 4:**
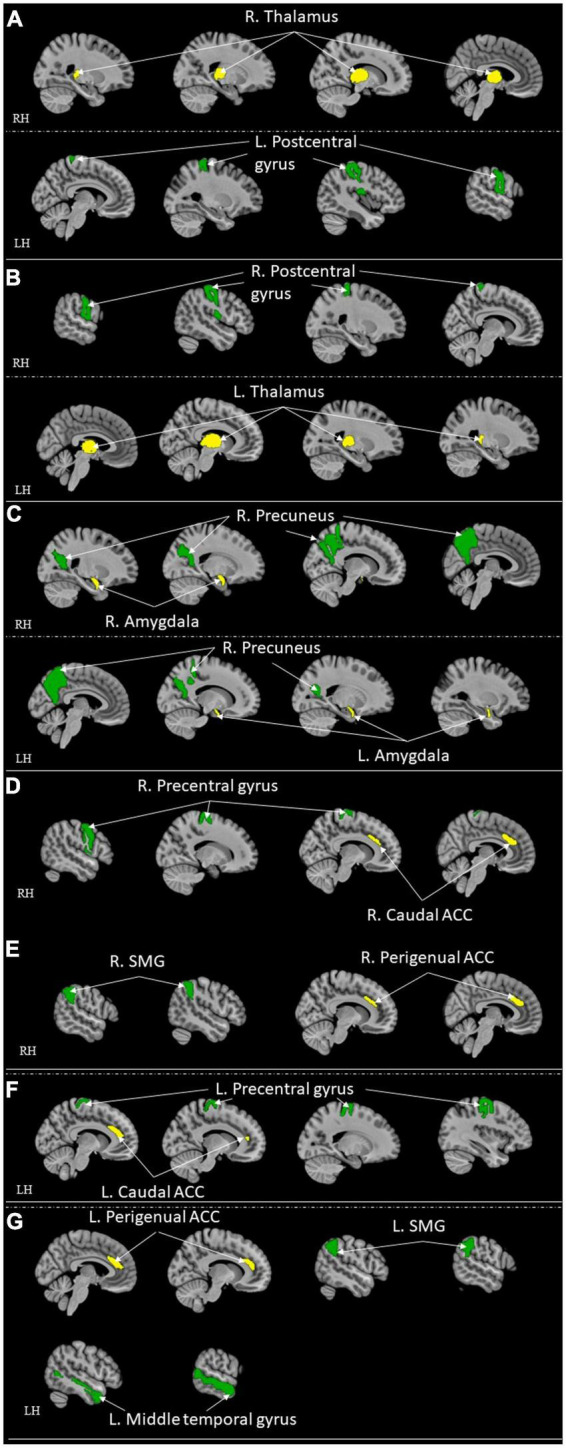
ROI-to-ROI functional connectivity for comparison studies: healthy control > high resilient individuals. Green represents the target regions. Yellow represents the seed regions. Each sub-section represents connectivity from a same seed: **(A)** R. Thalamus (Jeon et al., 2020); **(B)** L. Thalamus (Jeon et al., 2020); **(C)** Bilateral Amygdala (Singh et al., 2018); **(D)** R. Caudal ACC (Kennis et al., 2015); **(E)** R. Perigenual ACC (Kennis et al., 2015); **(F)** L. Caudal ACC (Kennis et al., 2015); **(G)** L. Perigenual ACC (Kennis et al., 2015). All highlighted regions showed in the figure are only used for approximate representation of the reported regions. It does not represent exact coordinates nor clusters of the reported results. R, Right; L, Left; RH, right hemisphere; LH, left hemisphere; ACC, anterior cingulate cortex; SMG, supramarginal gyrus.

**FIGURE 5:**
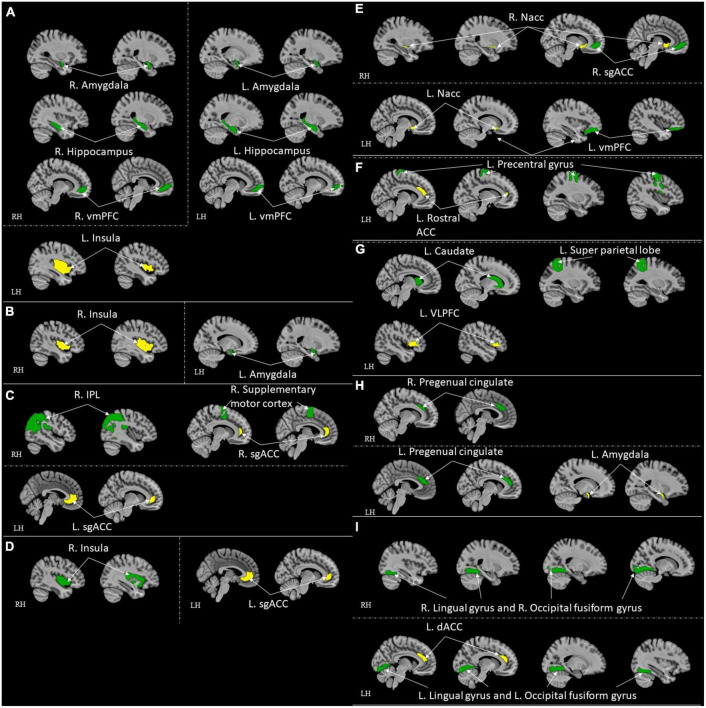
ROI-to-ROI functional connectivity for comparison studies: high resilient individuals > healthy control. Green represents the target regions. Yellow represents the seed regions. Each sub-section represents connectivity from a same seed: **(A)** L. Insula (Jeong et al., 2019); **(B)** R. Insula (Jeong et al., 2019); **(C)** Bilateral sgACC (Singh et al., 2014; Hirshfeld-Becker et al., 2019); **(D)** L. sgACC (Shao et al., 2018); **(E)** R. Nacc (Whittaker et al., 2018); **(F)** L. Rostral ACC (Kennis et al., 2015); **(G)** L. VLPFC (Singh et al., 2014); **(H)** L. Amygdala (Singh et al., 2014); **(I)** L. dACC (van der Werff et al., 2013b). All highlighted regions showed in the figure are only used for approximate representation of the reported regions. It does not represent exact coordinates nor clusters of the reported results. R, Right; L, Left; RH, right hemisphere; LH, left hemisphere; vmPFC, ventromedial prefrontal cortex; sgACC, subgenual anterior cingulate; IPL, inferior parietal lobule; ACC, anterior cingulate cortex; VLPFC, ventrolateral prefrontal cortex; dACC, dorsal ACC.

The rest of the nine studies were comparison studies (Table 6; Figures 4, 5). None of them investigated the local activation of different brain regions. Thus, the long-range functional connectivity was the focus of these nine studies. The average score of the replicability assessment among these nine studies was also close to the overall average (*M* = 4.45, SD = 1.59). The lowest scored research is in this group (score = 1) (Hirshfeld-Becker et al., 2019), indicating an incomplete report of the pre-processing steps for the neuroimaging data. All nine studies adopted the ROI-to-ROI approach for data analysis.

**TABLE 6:** ROI-to-ROI functional connectivity for comparison studies.

Studies	Operational definition of high resilient group	Seed (ROIs/whole brain)	Target regions (ROIs/whole brain)	Resultant brain area/regions (from seed to sink)		Network related					Replicability score*
				**HR > Ctrl**	**HR < Ctrl**	**DMN**	**CEN**	**SN**	**MN**	**EN**	
Jeon et al., 2020	Trauma-exposed healthy participants	Bilateral thalamus	Whole brain (seed-to-voxel)		Right thalamus and left postcentral gyrus					✓	4
					Left thalamus and right postcentral gyrus					✓	
Jeong et al., 2019	separated by occupation (firefighter)	dACC, bilateral anterior insula, vmPFC, bilateral amygdala and hippocampus	Same as seed	Left insula and bilateral amygdalae				✓		✓	6
				Left insula and bilateral hippocampi				✓	✓		
				Left insula and ventromedial prefrontal cortex (vmPFC)		✓		✓			
				Right insula and left amygdala				✓		✓	
Hirshfeld-Becker et al., 2019	At-risk (offspring of parents with a lifetime history of MDD) participants	6 regions: DMN: mPFC and PCC, subgenual anterior cingulate cortex (sgACC), left and right dlPFC, and left and right amygdala	Whole brain (seed-to-voxel)	Subgenual anterior cingulate (sgACC) and right inferior parietal lobule (IPL)/precentral gyrus			✓			✓	1
Shao et al., 2018	Connor-Davidson Resilience Scale (CD-RISC)	Bilateral sgACC	Whole brain (seed-to-voxel)	Left subgenual anterior cingulate (sgACC) to right insula				✓		✓	6
Singh et al., 2018	At-risk (offspring of parents with depression) participants	Bilateral amygdala and bilateral nucleus accumbens (Nacc)	Whole brain (seed-to-voxel)		Negative connectivity: Amygdala and precuneus	✓	✓			✓	4
Whittaker et al., 2018	At-risk (non-affected siblings of patients with BD) participants	Bilateral Nacc	Whole brain (seed-to-voxel)	Nucleus accumbens (Nacc) and ventromedial prefrontal cortex (subgenual anterior cingulate)		✓				✓	6
Kennis et al., 2015	Veterans without PTSD	Five bilateral seed points in the ACC were selected: Caudal, Dorsal, Rostral, Perigenual and Subgenual	Whole brain (seed-to-voxel)		Bilateral caudal ACC and bilateral precentral gyrus			✓			4
					Bilateral Perigenual ACC and bilateral superior medial gyrus (SMG)	✓		✓			
					Left Perigenual ACC and left middle temporal gyrus	✓		✓			
				The left rostral ACC and the left precentral/middle frontal gyrus			✓	✓			
Singh et al., 2014	Healthy offspring of a parent with BD	The dorsal and ventral DMN, bilateral executive control (ECN) networks, left and right amygdala, left and right Ventrolateral prefrontal cortex (VLPFC), and the subgenual ACC	The dorsal and ventral DMN, bilateral executive control (ECN) networks, left and right amygdala, left and right Ventrolateral prefrontal cortex (VLPFC), and the subgenual ACC	Left Ventrolateral prefrontal cortex (VLPFC) and left superior parietal lobule			✓				5
				Left amygdala and pregenual cingulate				✓		✓	
				Subgenual cingulate and right supplementary motor cortex				✓		✓	
				Left VLPFC and left caudate			✓				
van der Werff et al., 2013b	Experienced childhood maltreatment but scored negative on any DSM-IV axis-1 disorder	Left and right amygdala for limbic network, left and right dACC for salience network, PCC for the DMN and left mPFC	Left and right amygdala for limbic network, left and right dACC for salience network, PCC for the DMN and left mPFC	Negative connectivity: dorsal ACC and lingual gyrus; dorsal ACC and the occipital fusiform gyrus				✓			5
						Maximum score = 10					

DMN, Default Mode Network; CEN, Central Executive Network; SN, Salience Network; MN, Memory Network; EN, Emotional Network.

*A higher score indicates more information was provided in controlling imaging quality. See Table 2 for calculation components and process.

Different *a priori* ROIs were investigated among these nine studies (refer to Table 6 for ROIs details). Jeon et al. (2020) had bilateral thalamus as the two *a priori* ROIs. Jeong et al. (2019) chose seven *a priori* ROIs for their study. Hirshfeld-Becker et al. (2019) adopted six *a priori* ROIs. Shao et al. (2018) chose bilateral sgACC as two *a priori* ROIs. Four *a priori* ROIs were selected in Singh et al. (2018) study. Whittaker et al. (2018) took bilateral Nacc as two *a priori* ROIs. Kennis et al. (2015) had five bilateral *a priori* ROIs. Singh et al. (2018) adopted nine *a priori* ROIs. van der Werff et al. (2013b) took six *a priori* ROIs. The ACC, PCC, mPFC, and amygdala were the most used *a priori* ROIs across these nine studies.

The operational definitions of the high resilience group were different among all nine studies. One study used the score from a subjective scale, CD-RISC (Shao et al., 2018). Another study used high-stress occupation as an indicator (firefighter) (Jeong et al., 2019). The other seven studies in this group chose at-risk individuals, who are closely related to the clinical patient, without ever diagnosed any axis-1 disorders as an indicator of high resilience, and compared with normal random controls (van der Werff et al., 2013b; Singh et al., 2014, 2018; Kennis et al., 2015; Whittaker et al., 2018; Hirshfeld-Becker et al., 2019; Jeon et al., 2020).

Modalities were also different among these nine studies. Four studies utilized correlation maps and Fishers z-transform with second-level group jeon analysis (Kennis et al., 2015; Shao et al., 2018; Jeong et al., 2019; Jeon et al., 2020). One study adopted gICA and dual regression procedures for analyses (Singh et al., 2014). Another study utilized ROIs-based functional connectivity (Singh et al., 2018). Hirshfeld-Becker et al. (2019) investigate the data with general linear modal analyses. Whittaker et al. (2018) explored the voxel-wise differences in a two-sample *t*-test. Inverse transformation matrices were adopted in the study by van der Werff et al. (2013b).

Following were the specific seed to sink functional connectivity findings from each included study in this group. Jeon et al. (2020) found that the control had higher functional connectivity between the right thalamus and left post-central gyrus and the left thalamus and right post-central gyrus when compared with the high resilience group. Jeong et al. (2019) found that the high resilience group had higher functional connectivity from the left insula to the bilateral amygdalae, to the bilateral hippocampi, and to the vmPFC, and from the right insula to the left amygdala when compared to the control. Hirshfeld-Becker et al. (2019) found that the high resilience group had higher functional connectivity between sgACC and right inferior parietal lobule (IPL)/precentral gyrus when compared to the control. Shao et al. (2018) found that the high resilience group had higher functional connectivity from the sgACC to the right insula when compared to the control. Singh et al. (2018) found that the high resilience group had functional connectivity from the amygdala and precuneus when compared to the control. Whittaker et al. (2018) found that high resilience group had higher functional connectivity between the Nacc and ventromedial prefrontal cortex (sgACC) when compared to the control. Kennis et al. (2015) found that control had higher functional connectivity from bilateral caudal ACC to bilateral precentral gyrus, from bilateral perigenual ACC to bilateral superior medial gyrus (SMG, and left perigenual ACC to left middle temporal gyrus when compared to high resilience group. They also found that high resilience had higher functional connectivity from the left rACC to the left precentral/middle frontal gyrus when compared to control (Kennis et al., 2015). Singh et al. (2014) found that the high resilience group had higher functional connectivity between the left ventrolateral prefrontal cortex (VLPFC) and the left superior parietal lobule, the left amygdala, the pregenual cingulate, sgACC and the right supplementary motor cortex, the left VLPFC and the left caudate when compared to control. van der Werff et al. (2013b) found that the high resilience group had more negative connectivity between the lingual gyrus and the occipital fusiform gyrus when compared with the control group.

Four studies found resultant regions involved in DMN (Kennis et al., 2015; Singh et al., 2018; Whittaker et al., 2018; Jeong et al., 2019). CEN is involved in the resultant regions found in four different studies (Singh et al., 2014; Kennis et al., 2015; Hirshfeld-Becker et al., 2019). Four studies found resultant regions included in SN (van der Werff et al., 2013b; Singh et al., 2014; Shao et al., 2018; Jeong et al., 2019). Only Jeong et al. (2019) had resultant regions involved in MN. Eight out of nine studies had resulting areas involved in EN (Singh et al., 2014, 2018; Kennis et al., 2015; Shao et al., 2018; Whittaker et al., 2018; Hirshfeld-Becker et al., 2019; Jeong et al., 2019; Jeon et al., 2020). Notably, SN is the network involved in most connectivity compared to other networks.

## 4. Discussion

To our knowledge, this is the first systematic review to summarize the results from resting-state fMRI research focusing on the non-psychiatric high resilience group. The summarized neural findings across studies that adopted various operational definitions of resilience can inform the underlying neurological mechanisms of the positive aspects of resilience.

Under the group of correlational studies, OFC was negatively correlated with resilience, and it was the only shared local activation findings from more than one study (Kong et al., 2018; Wang et al., 2020). For long-range functional connectivity, most of the regions in the reported connectivity pathways for both the correlational group and comparison group are within EN (Singh et al., 2014, 2018; Kennis et al., 2015; Uchida et al., 2015; Santarnecchi et al., 2018; Shao et al., 2018; Whittaker et al., 2018; Hirshfeld-Becker et al., 2019; Jeong et al., 2019; Shi et al., 2019; Jeon et al., 2020). EN included regions like OFC, amygdala, hypothalamus, and hippocampus, etc. (Catalino et al., 2020). Align with the literature, these regions are correlated with resilience (Simeon et al., 2007; Yu and Zhang, 2007; Russo et al., 2011; Leaver et al., 2018).

**FIGURE 6:**
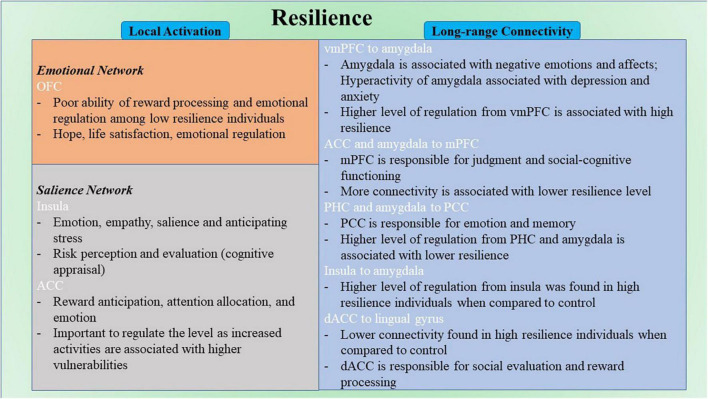
Schematic diagram for neural correlates of resilience. ACC, anterior cingulate cortex; dACC, dorsal anterior cingulate cortex; mPFC, medial prefrontal cortex; OFC, orbitofrontal cortex; PCC, posterior cingulate cortex; PHC, parahippocampal cortex; vmPFC, ventromedial prefrontal cortex.

Among correlational studies, only four studies explore the local activation of the brain and its relation to resilience. Notably, the activation in OFC was negatively correlated with resilience in two studies (Kong et al., 2018; Wang et al., 2020). Increased activation of this region has been found to be related to a number of stress-induced disorders (Bing et al., 2013; Liu et al., 2014; Xu et al., 2014; Qiu et al., 2015). However, the opposite effect was found in males specifically (Wang et al., 2020). The differences in hormonal systems and brain development between sex might cause sex-specific results (Wang et al., 2020). Gender should be considered as a vital factor when studying neuroimaging research in the future. Moreover, the OFC is involved in encoding reward value and emotional regulation (Berridge and Kringelbach, 2013; Shiba et al., 2016; Figure 6); it is a part of EN (Catalino et al., 2020). This finding might reflect the poor ability of reward processing and emotional regulation among low resilience individuals. The OFC is found to be closely related to hope (Wang et al., 2017), life satisfaction (Kong et al., 2015), emotion regulation (Wager et al., 2008; Berridge and Kringelbach, 2013; Shiba et al., 2016) and resilience (Simeon et al., 2007; Yu and Zhang, 2007), in which hope, life satisfaction, and emotion regulation are highly associated with resilience. For instance, hope and resilience have been found to be strongly and significantly related to each other (Hidayat and Nurhayati, 2019; Myers et al., 2019). Hope was found to be positively correlated with resilience and a significant predictor for resilience (Hidayat and Nurhayati, 2019; Myers et al., 2019). When paired together, hope and resilience were significant predictors of quality of life and wellbeing (Kirmani et al., 2015; Li et al., 2016; Long et al., 2020). Besides, emotional regulation strategies were essential for promoting resilience and preventing stress (Tugade and Fredrickson, 2007; Thomas and Zolkoski, 2020). It has been reported that better resilience often leads to better life satisfaction (Samani et al., 2007; Abolghasemi and Taklavi Varaniyab, 2010; Moradi and Mirkohi, 2020). These findings indicate the functional role of OFC in resilience, and appropriate regulation of the functioning of OFC from regions like dlPFC (Golkar et al., 2012) could be a potential feature of high resilience. However, with limited studies in this part, this finding may need further validation in future research with large sample size and a more standard measure of resilience. From the limited results for local activation included in this review, there is no common region across different resilience measures. Both studies that found the correlation between OFC and resilience had CD-RISC as their operational definition of resilience (Kong et al., 2018; Wang et al., 2020). Other regions such as rPFC, SPL, ACC, and insula were sparsely reported across the included studies (Fujisawa et al., 2015; Kong et al., 2015). For instance, the left rostral prefrontal cortex and superior parietal lobule were found to associate positively with resilience (Fujisawa et al., 2015) these regions are mainly responsible for complex cognitive functioning, including memory, problem-solving, judgment, and perception (Burgess et al., 2007; Volle et al., 2011). In another study, the ACC and insula were also reported to negatively correlate with resilience (Kong et al., 2015). The ACC is mainly responsible for higher-level functions such as reward anticipation, attention allocation, and emotion (Pardo et al., 1990; Bush et al., 2002; Decety and Jackson, 2004; Figure 6). The insula is primarily responsible for emotions (Phan et al., 2002; Vilares et al., 2012; Figure 6). From previous literature, insula was found to be involved in risk perception and evaluation (Zhou et al., 2014; Lau et al., 2015). These perception and evaluation are one form of cognitive appraisal, which is one of the key factors that influencing resilience (Hooberman et al., 2010; Kalisch et al., 2015).

The other seven studies in the correlational group explored the long-range functional connectivity and its relation to resilience. Four out of seven studies took a ROI-to-ROI approach. With differences among the *a priori* ROIs and MRI modality, there was no common pathway that was correlated with resilience between multiple studies. However, the regions in EN are the most involved in the reported connectivity pathways (Uchida et al., 2015; Santarnecchi et al., 2018; Shi et al., 2019). The brain regions include the amygdala and OFC. This result highlighted the importance of emotional regulation in resilience. The amygdala was found to be linked to negative emotions and affects (Murray, 2007; Figure 6). Hyperactivity of the amygdala was shown to be associated with depression and anxiety (Linden, 2006). The connectivity strength from left amygdala to left precuneus was found to be positively associated with depressive symptoms in individuals with subthreshold depression individuals (Peng et al., 2020). On the other hand, high resilience is associated with lower amygdala function (Leaver et al., 2018). A higher level of regulation of the functional activity of the amygdala from other brain regions like the vmPFC (Motzkin et al., 2015; Figure 6) would be one of the keys to staying resilient. In addition, regions in DMN were reported across most studies in this group (Kilpatrick et al., 2015; Uchida et al., 2015; Santarnecchi et al., 2018; Miyagi et al., 2020; Shi et al., 2021). The DMN is found to be active during rest and mind-wandering (Buckner et al., 2008; Andrews-Hanna, 2012). Although more studies reported regions that included the DMN, more pathways were reported that involved regions in EN. Regions like mPFC and PCC were included in this network. The pathways from ACC and right amygdala to mPFC were found to be negatively correlated to resilience (Uchida et al., 2015; Santarnecchi et al., 2018). The mPFC is mainly responsible for judgment and social-cognitive functioning (Mitchell et al., 2002; Figure 6). The pathways from the right parahippocampal cortex (PHC) and the right amygdala to PCC were found to be negatively correlated to resilience (Uchida et al., 2015; Miyagi et al., 2020). The PCC is mainly responsible for emotion and memory (Maddock et al., 2001, 2003; Figure 6). All seven included studies in this group used different resilience measures. However, no same pathways were reported from different studies in this group. There is a high possibility that different modality and different operational definitions of resilience target different neural resilience mechanisms, leading to various results across studies. Other regions such as PHC and angular gyrus were also reported in different pathways correlated with resilience. The pathway from the right PHC to the left retrosplenial cortex and PCC was found to be negatively correlated with resilience (Miyagi et al., 2020). However, the pathway from the left insula to the right PHG was found to be positively correlated with resilience (Shi et al., 2019). The PHG is responsible for scene recognition (Aguirre et al., 1998; Epstein and Kanwisher, 1998). The pathway between the left angular gyrus and the bilateral occipital pole was found to be positively correlated with resilience (Santarnecchi et al., 2018). The angular gyrus is mainly responsible for attention and memory retrieval (Park et al., 2008; Seghier, 2013).

Among comparison studies, a similar pattern was found in these nine studies compared with the seven studies in the correlational studies group that explored the long-range functional connectivity. Similarly, because all nine studies took a ROI-to-ROI approach, the connectivity results were mixed. However, seven out of nine studies found resultant pathways that included the amygdala and ACC (Singh et al., 2014; Kennis et al., 2015; Shao et al., 2018; Whittaker et al., 2018; Hirshfeld-Becker et al., 2019; Jeong et al., 2019). Eight out of nine studies had found brain regions involved in EN (Singh et al., 2014, 2018; Kennis et al., 2015; Shao et al., 2018; Whittaker et al., 2018; Hirshfeld-Becker et al., 2019; Jeong et al., 2019; Jeon et al., 2020). Among all the resultant pathways, 18 had regions involved in EN. Specifically, the ability to regulate emotions through SN–EN connectivity within the high resilience group was better compared with control. The pathways that involved regions in SN were also reported in four studies (van der Werff et al., 2013b; Singh et al., 2014; Shao et al., 2018; Jeong et al., 2019). The SN is found to be mainly involved in detecting changes in sensory stimuli (Downar et al., 2000), and modulating the switch between DMN and the central executive network (Menon, 2015). Regions reported in SN include insula and dACC. The connectivity from left insula to vmPFC, bilateral amygdalae and hippocampi, and right insula to left amygdala were found to be more in high resilience individuals compared to control (Jeong et al., 2019). The connectivity from the left sgACC to right insula was also found to be higher in high resilience individuals when compared with control (Shao et al., 2018). The insula is mainly responsible for empathy (Singer, 2006), emotions (Phan et al., 2002), and salience (Taylor et al., 2009; Menon and Uddin, 2010; Figure 6). The connectivity from left dACC to the bilateral lingual gyrus and the occipital fusiform gyrus was found to be lower among high resilience individuals compared with control (van der Werff et al., 2013b). The dACC is mainly responsible for social evaluation (Dedovic et al., 2016) and reward processing (Bush et al., 2002; Figure 6). Mostly reported regions within these pathways included the amygdala, the insula, and the ACC. Insula is involved in a number of complex functioning, including anticipation of stress (Simmons et al., 2012). Activation of this region were found to be among high resilient individuals when presented with aversive stimuli (Waugh et al., 2008). Align with the summarized result from the correlational group and previous literature, emotion regulation is highly associated with resilience (Karreman and Vingerhoets, 2012; Mestre et al., 2017; Polizzi and Lynn, 2021). The ACC is the most reported region that included studies in this group. Similar to the amygdala, increased activities of ACC are associated with higher vulnerabilities (Bolsinger et al., 2018). Therefore, it is also important to regulate the level of the functional activity of ACC to maintain a high resilience level. Similar to the studies in the correlational group, different operational definitions were used, and no common pathway was found among all included studies in this group. Other regions such as amygdala and ACC were also in individual studies (Singh et al., 2014, 2018; Kennis et al., 2015; Shao et al., 2018; Hirshfeld-Becker et al., 2019; Jeong et al., 2019).

Among all 19 studies, the CD-RISC was the most commonly used scale for defining resilience. A total of five studies used this scale to operationally define resilience (Kong et al., 2015; Shao et al., 2018; Miyagi et al., 2020; Wang et al., 2020). Two out of these five studies found a similar relationship between OFC and resilience (Kong et al., 2018; Wang et al., 2020). In two of these five studies, ACC was found to be included in the resultant regions that related to resilience (Kong et al., 2015; Shao et al., 2018). Both OFC and ACC are included in the EN, indicating the critical role of emotional regulation on resilience (Catalino et al., 2020). Although seven studies used the absence of psychopathologies as an indicator of resilience, the types of disorders vary (van der Werff et al., 2013b; Singh et al., 2014, 2018; Kennis et al., 2015; Whittaker et al., 2018; Hirshfeld-Becker et al., 2019; Jeon et al., 2020). This explained the result difference among these seven studies. The rest of the studies adopted different operational definitions of resilience and explained the various results summarized in this review. Even studies adopted same operational definitions of resilience, different neural feature were found in relation to resilience. The demographic of the participants and the methodological difference may cause the differences. When more studies examined the same operational definitions of resilience, a meta-analysis can be conducted for a better understanding of this difference.

The existing neural model for vulnerability and resilience (Homberg and Jagiellowicz, 2022) pointed the importance of attention shifting and cognitive flexibility. The connectivity between the PCC and the vmPFC was found to be associated with cognitive flexibility (Lau et al., 2020). Yet, this connection was not found in the included studies in this review, as for attention shifting.

The summarized results from this review align with the resting-state fMRI findings from other similar reviews in resilience and neurological research. Bolsinger et al. (2018) conducted similar review targeting adults who experienced traumatic events. Similar to the finding from this review, they also found the relationship between the amygdala, ACC, and resilience. Similar results of ACC were also indicated in the review by van der Werff et al. (2013b). They reviewed structural, resting-state, and task-based neuroimaging studies of resilience in adults. Holz et al. (2020) reviewed the relationship between environmental factors, neurological mechanisms, and resilience. They also found the involvement of the brain area in reward processing associated with resilience. Lastly, Eaton et al. (2022) conducted a review targeting the brain’s structure, function and connectivity, and resilience in youth. Notably, Eaton et al. (2022) targeted studies with a younger population only, which excluded most of the studies included in the current review. In the findings of fMRI studies in their review, areas involved in reward processing and emotional regulation were suggested to be associated with resilience. In the review from Eaton et al. (2022), the included task-based studies also found similar resultant regions, like PFC and amygdala, and functions regarding emotional regulation and reward processing (Heitzeg et al., 2008; Hanson et al., 2015; Luking et al., 2018; Rodman et al., 2019). Lower amygdala responses to negative stimuli, and tighter coupling of a PFC-amygdala circuit were found among high resilience individuals in task-based studies (Heitzeg et al., 2008; Rodman et al., 2019; Eaton et al., 2022). One of the studies in the current review also found that the right amygdala to mPFC connectivity was negatively associated with resilience (Uchida et al., 2015). This linked the findings between task-based studies and rest-state studies on resilience; however, there were no other studies had similar results. Align with similar reviews in resilience, similar findings on the association between emotion regulation, reward processing, and resilience were found. A suitable strategy for regulating emotions is essential for promoting resilience (Tugade and Fredrickson, 2007). Emotional regulation is also one of the main focuses of preventive interventions promoting resilience (Greenberg, 2006). Properly regulating emotion is one of the key protective factors of resilience (Troy and Mauss, 2011). On the other hand, hypoactivity of the OFC was associated with a decreased level of dopamine receptors (Volkow et al., 2002). The dopamine system plays a vital role in mediating reward processing (Stein, 2009). The association between OFC and the dopamine system suggests that hypoactivity of the OFC may influence the reward system via the dopamine system. In addition, Reward stimuli were found to have a buffering effect on stress (Dutcher and Creswell, 2018). Summarized from previous literature and the results from the included studies in this review, reward processing is also one of the key protective factors of resilience.

## 5. Limitations

Some limitations should be noted in this review. First, no quantitative analysis can be performed due to the limited studies included in each category. Although there were 19 included papers, the study designs were different, resulted in only nine to ten papers per category. In addition, this review adopted strict inclusion criteria. Only papers that clearly defined resilience were included. Resilience is a board topic when considering from different aspects. By limiting the focus, it helps this review to focus on the main purpose of assessing the neural correlates of resilience among mentally healthy individuals, and the protective mechanisms of resilience. Second, among the included studies, the average score for replicability was below 50% of the total score (*M* = 4.42). This score indicated a limit to the robustness of the results reported from each of the included study. With the lack of the control of the fMRI data or failed to report on the pre-processing steps of the data, it led to a concern of the trustworthiness of the results published. Replicability has been a concern among the field of resting-state fMRI studies (Chen X. et al., 2018). Future studies are suggested to account for and to report proper processing steps for imaging data for the robustness of the results. Third, reverse inference could be an issue for resting-state fMRI studies. Reasons backward from the neural activation to a cognitive function is a common concern over the interpretation of resting-state fMRI findings in the field of cognitive neuroscience (Poldrack, 2006; Wager et al., 2016). However, there is no common task that is universally accepted for assessing resilience in task-based fMRI. As mentioned in this review, inconsistent task, inconsistent difficulties, and variation on individual abilities limited the interpretation of neural result in relation to resilience. Since this review focused on resting-state fMRI studies, there are no specific cognitive process engaged. The inferences made by the findings in other relating neuroimaging studies with specific cognitive process in question might not be a major concern for resilience research. Anyhow, combination of resting-state fMRI and task-based fMRI, e.g., by adopting a naturalistic paradigm is recommended for future resilience neuroimaging studies. Future studies should also carefully consider the selection of brain regions of interests and the probability of the cognitive process in question in order to improve the confidence in reverse inferences (Poldrack, 2006). Lastly, as mentioned in the introduction, other neurological factors like neuroendocrine and monoamines can affect resilience-dependent change in neural activities captured in resting-state fMRI (Russo et al., 2012; Watanabe and Takeda, 2022). While this review is primarily focused on the resting-state studies in relation to resilience, future studies are suggested to include other neurological factors that may cause resilience-dependent change in neural activities.

## 6. Conclusion

This systematic review explored the resting-state neural correlates of resilience among high resilience individuals. Based on the findings, low resting-state activity of ACC, amygdala, and OFC and high resting-state insula activity could be the potential neural feature of high resilient. Brain regions involved in reward processing and emotional regulation were found in multiple studies associated with resilience. This result highlighted the importance of strategies for regulating emotions and perceiving rewards to enhance resilience. Future neuroimaging studies on resilience should consider adopting multiple resting-state fMRI modalities as well as operational definitions of resilience for plausible meta-analysis.

## Author contributions

AT: study concept and design, data collection, data analysis, interpretation, and writing the manuscript. M-KL and XG: data interpretation and reviewing the manuscript. WL: study concept and design, data analysis, interpretation, and reviewing the manuscript. All authors contributed to the article and approved the submitted version.

## Footnotes

1
www.brainmap.org
